# Pharmacist-led hospital intervention reduces unintentional patient-generated medication discrepancies after hospital discharge

**DOI:** 10.3389/fphar.2024.1483932

**Published:** 2024-10-24

**Authors:** Maja Jošt, Lea Knez, Mitja Kos, Mojca Kerec Kos

**Affiliations:** ^1^ Pharmacy, University Clinic Golnik, Golnik, Slovenia; ^2^ Faculty of Pharmacy, University of Ljubljana, Ljubljana, Slovenia

**Keywords:** medication reconciliation, patient counselling, pharmacist-led intervention, transition of care, post-discharge therapy, medication discrepancies, patient-generated changes

## Abstract

**Background:**

Medication reconciliation can significantly reduce clinically important medication errors at hospital discharge, but its impact on post-discharge medication management has not been investigated. We aimed to investigate the incidence of patient-generated medication discrepancies 30 days after hospital discharge and the impact of a pharmacist-led medication reconciliation coupled with patient counselling on clinically important discrepancies caused by patients.

**Methods:**

A pragmatic, prospective, controlled clinical trial was conducted at the University Clinic Golnik, Slovenia. Adult patients were divided into an intervention group and a control group. The intervention group received pharmacist-led medication reconciliation at admission and discharge, plus patient counselling at discharge. Medication discrepancies were identified by comparing the therapy prescribed in the discharge letters with the therapy 30 days after discharge, obtained through telephone patient interviews. Discrepancies were classified as intentional or unintentional, and their clinical importance was assessed.

**Results:**

The study included 254 patients (57.9% male, median age 71 years), with 136 in the intervention group and 118 in the control group. Discrepancies occurred with a quarter of the medicines (617/2,441; 25.3%) at 30 days after hospital discharge, and patients themselves caused half of the discrepancies (323/617; 52.4%), either intentionally (171/617; 27.7%) or unintentionally (152/617; 24.6%). Clinically important discrepancies occurred in 18.7% of intentional and 45.4% of unintentional patient-generated changes. The intervention significantly reduced the likelihood of clinically important unintentional patient-generated discrepancies (OR 0.204; 95%CI: 0.093–0.448), but not clinically important intentional patient-generated discrepancies (OR 2.525; 95%CI: 0.843–7.563). The latter were more frequent among younger, male patients and patients hospitalized for respiratory diseases.

**Conclusion:**

The study emphasizes the importance of addressing discrepancies made by patients after hospital discharge, which can result in potentially harmful outcomes. It also shows that a pharmacist-led hospital intervention can significantly reduce discrepancies in the early post-discharge period. These findings can guide the development of future services to improve patient support for medication management after hospitalization.

## 1 Introduction

Transitions of care, particularly those involving hospitalizations, are associated with significant changes in a patient’s health status, functional abilities, and therapeutic regimen. Changes in medicines occur in virtually every patient at hospital discharge and such changes often constitute a medication error (Jošt et al., 2024; Viktil et al., 2012). More than 60% of patients experiencing an unintentional discrepancy or an undocumented intentional discrepancy when discharged from a hospital (Jošt et al., 2024). These medication errors may persist long after hospital discharge (Viktil et al., 2012) and increase patients’ risk of hospital readmissions and other healthcare utilization (Coleman et al., 2005; Forster et al., 2005; Uitvlugt et al., 2022).

The detrimental effects of medication errors that occur at hospital discharge may escalate after patients return home, as the responsibility for managing medicines shifts more heavily to patients or their caregivers (Kardas et al., 2013). Furthermore, the increased complexity of drug therapy after hospital discharge makes medication management even more challenging for patients, often worsening their already compromised functional status.

General practitioners (GPs) have a crucial role in overseeing and coordinating patient care after hospital discharge, but they often receive delayed or limited information about patient transitions, which can disrupt continuous care (Karapinar et al., 2010). Thus, interventions such as medication reconciliation that are known to reduce medication errors at transitions of care (Jošt et al., 2024; Schnipper et al., 2022) should be designed to empower both patients and GPs. The involvement of pharmacists in these interventions can improve patients’ ability to manage their medicines and support GPs in maintaining seamless, informed care coordination (Marinović et al., 2021; Ravn-Nielsen et al., 2018; Stroud et al., 2019).

Communication is vital in the efficient transfer of information between healthcare professionals, patients, and caregivers. Several interventions have been developed to improve communication at hospital discharge and increase medication safety during transitions of care. Notably, patient education and medication counselling have been shown to play an important role in the success of interventions based on medication reconciliation (Dautzenberg et al., 2021). Furthermore, patients were found to adhere better to therapy when they were involved in decision-making (Kardas, 2024).

Despite challenges expected in medication management after hospital discharge, there has been little research on the topic. In fact, most studies on medication reconciliation focus on the discharge process itself, with limited data on the persistence of medication errors or the occurrence of new errors during the initial post-discharge period. Greater insight on such errors, especially the patient’s role in them, is essential to further improve medication reconciliation and related services, which would potentially enhance patient outcomes and also reduce healthcare utilization.

Therefore, our aim in this study was to evaluate the incidence of patient-generated medication discrepancies 30 days after hospital discharge and to examine the impact of a pharmacist-led hospital intervention on clinically important intentional and unintentional medication discrepancies caused by patients.

## 2 Materials and methods

A pragmatic, prospective, controlled clinical trial was conducted at the University Clinic Golnik, Slovenia. Adult medical patients were allocated into the intervention or control (standard care) group. The intervention included a pharmacist-led medication reconciliation at admission and discharge and patient counselling at hospital discharge. No additional intervention was carried out after hospital discharge for either group. Patients were interview by telephone 30 days after discharge, and medication discrepancies were determined by comparing the therapy they were using at that point with the therapy detailed in the discharge letter. The discrepancies made by the patients were classified as intentional or unintentional, and their clinical importance was assessed.

This study was conducted within the context of a clinical trial evaluating the effectiveness of pharmacist-led medication reconciliation on medication errors at hospital discharge and healthcare utilization in the next 30 days and the methods of this study are described in details elsewhere (Jošt et al., 2024).

All procedures were conducted in accordance with institutional and national ethical standards and with the 1975 Declaration of Helsinki and its subsequent amendments or comparable ethical standards. The study was approved by the National Medical Ethics Committee of the Republic of Slovenia (protocol number 0120-223/2019/4) and registered at ClinicalTrials.gov (NCT06207500). Informed consent was obtained from all patients included in this study.

### 2.1 Inclusion of study participants

The trial enrolled adult medical patients from five general medical wards who were hospitalized at the University Clinic Golnik in Slovenia. At hospital admission, the allocation of patients in one of the five wards was random, depending on the availability of beds. The intervention group comprised patients consecutively admitted to the ward where medication reconciliation was integrated into routine clinical practice. The control group consisted of randomly selected patients from the remaining four wards, using Research Randomizer (Urbaniak and Pious 2011) and following the inclusion pace in the intervention group. Patients and healthcare staff were not blinded and the study was run as pragmatic trial.

After inclusion in the study, patients may have been subsequently excluded if they were hospitalized for diagnostic purposes only, transferred to another ward or hospital, offered medication reconciliation in the control group, or died during hospitalization.

### 2.2 Data collection

Data collection and outcome assessment were performed by research clinical pharmacists who were not involved in the treatment of included patients. Researchers were trained according to standard operating procedures which included practical examples to make the assessment more objective. In case of difficulties, the research pharmacists consulted each other.

The data were collected from patients’ medical records and study documentation. A telephone interview with patients or caregivers was undertaken by the research pharmacist within 30 (±5) days after discharge from hospital.

Patients’ comorbidities were assessed using the Charlson Comorbidity Index (Quan et al., 2005; Shebeshi et al., 2021). The reason for a patient’s index hospital admission was obtained from the discharge letter and categorized as an acute or planned admission. Medicines were grouped into the nine most common classes of medicines: medicines for respiratory disease (ATC code R03); antihypertensives (ATC codes C07-09); antithrombotic medicines (ATC code B01); analgesics (ATC code N02); medicines for acid-related disorders (ATC code A02); sedatives, antidepressants, and antipsychotics (ATC code N05-06); antidiabetics (ATC code A10); diuretics in heart failure (ATC code C03C-03D); and other medicines. Discrepancies at discharge were determined by comparing the best possible medication history with the medicines in the discharge letter. Discrepancies at discharge were further classified as *unintentional*, *undocumented intentional*, or *documented intentional*. All unintentional and undocumented intentional discrepancies were defined as medication errors.

The primary outcome of the study was defined as clinically important patient-generated medication discrepancies 30 days after hospital discharge. The discrepancies were obtained by comparing the therapy a patient was taking 30 days after discharge, as recorded via the telephone interview, with medicines in the discharge letter. Discrepancies were classified as *no discrepancy*, *change in dosing regimen*, *omission*, or *addition*. Based on information provided by patients via the phone interview, discrepancies at 30 days after discharge were classified as *GP-generated* (if the change was made by the GP), *specialist-generated* (if the change was made by the specialist), *intentional patient-generated* (if the change was made intentionally by the patient), and *unintentional patient-generated* (if the change was made unintentionally by the patient).

Clinical importance of patient-generated discrepancies was rated using a 4-point Likert scale to describe the *medication* errors: 1 = *not important*, 2 = *not very important*, 3 = *very important*, and 4 = *life-threatening*. Very important and life-threatening discrepancies represented *clinically important discrepancies*.

### 2.3 Statistical analysis

Multiple logistic regression was employed to examine the impact of the intervention and other cofactors, such as sex, age, number of medicines at discharge, comorbidities, and type of and reason for admission, on clinically important patient-generated medication discrepancies at 30 days after discharge. Model fit was evaluated using the Hosmer–Lemeshow goodness-of-fit test. Nagelkerke’s R^2^ was used to gain insight into the model’s explanatory power. The significance of individual variables was analysed by the Wald statistical test. The results are presented as odds ratios (ORs) with 95% confidence intervals (CIs).

The sample size was predicted based on the rule of thumb used to define the sample size for the purpose of multiple logistic regression. This assumes 10 “events” for each exposure factor. In our case, an event was defined as a case of a patient with at least one clinically important discrepancy observed 30 days after hospital discharge. For the purpose of the study planning, we assumed 30%–50% of the patients would present with intentional or nonintentional patient-generated discrepancy. With the statistical model, we intended to use approximately 10 predictive factors. Therefore, the rule-of-thumb sample prediction required approximately 200–300 patients.

Descriptive statistics were used to describe the baseline characteristics of the participants. Apart from the multivariable logistic regressions, a univariable statistical analysis was performed for specific comparisons between the intervention and control groups. The chi-square test or Fisher’s exact probability test was used for categorical variables, and the nonparametric Mann‒Whitney U test for continuous variables.

All the statistical analyses were performed using the statistical program IBM SPSS Statistics version 28.0. A significance level of α = 0.05 was used for all tests.

## 3 Results

A total of 254 patients—136 in the intervention group and 118 in the control group—were included in the analysis of medication discrepancies 30 days after hospital discharge. The study population included slightly more male patients (57.9%), older adult patients (median 71 years; IQR 63-79), and polymorbid patients (median Charlson Comorbidity Index of 2, IQR 1-4) who were taking a median of six medicines prior to hospital admission. The baseline characteristics were similar in both groups (Table 1); however, the groups differed in some characteristics of the index hospitalization. In the intervention group, more patients were admitted for an acute reason (91.2% vs. 66.4%, *p* < 0.001) and had a shorter hospital stay (5.5 vs. 8 days, *p* < 0.001) than in the control group. The reasons for admission also differed between the groups. In the intervention group, significantly more patients were admitted for infections and fewer for malignancies and respiratory diseases.

**TABLE 1:** Patients’ characteristics.

	All patients		Intervention group		Control group		*p*-value
	N = 254		N = 136		N = 118		
Gender; male (n, %)	147	57.9%	86	63.3%	61	51.7%	0.063*
Age (years; median, IQR)	71	(63–79)	72	(63–79)	70	(63–73)	0.485**
Charlson Comorbidity Index (median, IQR)	2	(1–4)	2	(1–3)	2	(1–4)	0.236**
Admission type; acute (n, %)	200	78.7%	124	91.2%	76	66.4%	<0.001*
Reason for admission (n, %)							
Infection	88	34.6%	61	44.9%	27	22.9%	0.003*
Respiratory disease	61	24.2%	26	19.1%	35	29.7%	
Heart disease	43	16.9%	23	16.9%	20	16.9%	
Malignancy	36	14.1%	13	9.6%	23	19.5%	
Other	26	10.2%	13	9.6%	13	11.0%	
Duration of hospitalization (days; median, IQR)	7	(4–10)	5.5	(4–9)	8	(6–13)	<0.001**
Number of medicines on admission (median, IQR)	6	(3–9)	6	(3–10)	7	(4–9)	0.785**
Number of medicines on discharge (median, IQR)	7	(4–10)	8	(5–10)	7	(4–9)	0.087**

Abbreviations: IQR, interquartile range; significant *p* values are marked in bold. *Chi square test; **Mann‒Whitney U test.

Among the 254 patients, the total number of medicines increased from 1,898 at discharge to 2,441 at 30 days after discharge. Despite this increase, the median number of medicines per patient remained unchanged at seven (IQR 4-10), with no significant difference between the groups. Patients were most frequently treated with medicines for respiratory diseases (15.0%) and antihypertensive medicines (14.8%) followed by antithrombotic medicines (7.3%) and analgesics (7.3%) (Table 2).

**TABLE 2:** Medicines and medication discrepancies 30 days after discharge.

	Number of medicines					
	All patients N = 2,441		Intervention group N = 1,265		Control group N = 1,176	
Medicine classes (n, %)						
Drugs for respiratory diseases	365	15.0%	173	13.7%	192	16.3%
Antihypertensives	362	14.8%	205	16.2%	157	13.4%
Antithrombotic	179	7.3%	93	7.4%	86	7.3%
Analgesics	178	7.3%	75	5.9%	103	8.8%
Drugs for acid related disorders	154	6.3%	72	5.7%	82	7.0%
Sedatives, antidepressants and antipsychotics	130	5.3%	69	5.5%	61	5.2%
Antidiabetics	108	4.4%	64	5.1%	44	3.7%
Diuretics	99	4.1%	57	4.5%	42	3.6%
Other	866	35.5%	457	36.1%	409	34.8%
Discrepancy type (n, %)						
NO discrepancy	1824	74.7%	1,033	81.7%	791	67.3%
Discrepancy	617	25.3%	232	18.3%	385	32.7%
• Change in dosage regimen	187	7.7%	87	6.9%	100	8.5%
• Omission	136	5.6%	55	4.3%	81	6.9%
• Addition	294	12.0%	90	7.1%	204	17.3%
Discrepancy generated by						
NO discrepancy	1,824	74.7%	1,033	81.7%	791	67.3%
Patient-intentional	171	7.0%	71	5.6%	100	8.5%
• Clinically important	32	1.3%	20	1.6%	12	1.0%
Patient-unintentional	152	6.2%	26	2.1%	126	10.7%
• Clinically important	69	2.8%	16	1.3%	53	4.5%
General practitioner	126	5.2%	60	4.7%	66	5.6%
Specialist	168	6.9%	75	5.9%	93	7.9%

Overall, a quarter of medicines (617/2,441; 25.3%) had a discrepancy 30 days after hospital discharge (Table 2). The most common discrepancies were additions of medicines (294/2,441; 12.0%), followed by changes in dosage regimens (187/2,441; 7.7%) and, less frequently, omissions (136/2,441; 5.6%). Approximately one-third of the discrepancies (232/617 37.6%) appeared for the first time 30 days after discharge, either as addition of a new medicine (147/617; 23.8%), an omission (45/617; 7.3%), or a change in dosage regimen (40/617; 6.5%). The remaining two-thirds of the discrepancies (385/617; 62.4%) occurred with medicines that already showed discrepancies at discharge. The discrepancies 30 days after discharge were often the opposite of the discrepancies that occurred at discharge; for example, an omission of a medicine at discharge was followed by a medicine addition and vice versa (Supplementary Table S1).

The detected discrepancies were generated to a similar extent by patients (323/2,441; 13.2%) and physicians (294/2,441; 12.1%). Interestingly, patient-generated intentional and unintentional discrepancies had approximately the same incidence (171/2,441; 7.0% and 152/2,441; 6.2%, respectively). Patient-generated unintentional discrepancies were more often due to changes in dosage regimens (66/152; 35.3%) or medicine omissions (42/152; 30.9%), while patient-generated intentional discrepancies were evenly distributed among all discrepancy types (Table 3). Most discrepancies generated by patients involved medicines for pulmonary diseases and acid-related disorders, both unintentionally and intentionally. Patient-generated intentional discrepancies also frequently occurred for analgesics and sedatives (Supplementary Table S2).Over half of the medication errors arising at hospital discharge (296/526; 56.3%) were sustained at 30 days after discharge, the rest being more often changed or possibly resolved by the patient, either intentionally (88/526; 16.7%) or unintentionally (94/526; 17.9%), and only to a lesser extent by GPs and specialists (48/526; 9.1%; Chi-square, *p* < 0.001; Supplementary Table S3).

**TABLE 3:** Person generating discrepancy vs. type of discrepancy 30 days after discharge (N = 617).

		Person generating discrepancy 30 days after discharge			
		Physician (N = 294)	Patient-intentional (N = 171)	Patient-unintentional (N = 152)	*p*-value*
Discrepancy type	Change in dosage regimen (N = 187)	62 (33.2%)	59 (31.6%)	66 (35.3%)	<0.001
	Omission (N = 136)	59 (43.4%)	35 (25.7%)	42 (30.9%)	
	Addition (N = 294)	173 (58.8%)	77 (26.2%)	44 (15.0%)	

*Chi square test.

The majority of patients (81.5%) had at least one discrepancy between the instructions in the discharge letter and the therapy at 30 days after discharge (Table 4). However, in the intervention group significantly fewer patients experienced at least one discrepancy (74.3% vs. 89.8%; *p* < 0.001) and the median number of discrepancies per patient was lower than in the control group (0; IQR 0-1 vs. 1.5; IQR 0-3; *p* < 0.001).

**TABLE 4:** Patients with medication discrepancies at 30 days after discharge.

	All patients N = 254		Intervention group N = 136		Control group N = 118		*p*-*value*
Patients with a discrepancy (n, %)	207	81.5%	101	74.3%	106	89.8%	0.001*
Patients with unintentional patient-generated discrepancy (n, %)	90	35.4%	20	14.7%	70	59.3%	<0.001*
• Clinically important discrepancy (n, %)	49	19.3%	11	8.1%	38	32.2%	<0.001*
Patients with intentional patient-generated discrepancy (n, %)	106	41.7%	50	36.8%	56	47.5%	0.085*
• Clinically important discrepancy (n, %)	24	9.4%	15	11.0%	9	7.6%	0.355*
Patients with clinically important patient-generated discrepancy (n, %)*^#^	67	26.3%	25	18.4%	42	35.6%	0.002*
Number of discrepancies; median (IQR)	1	(0–2)	0	(0–1)	1.5	(0–3)	<0.001**

Abbreviations: IQR, interquartile range; significant *p* values are marked in bold. *Chi square test; **Mann‒Whitney U test.

^#^Patients with ANY clinical important patient-generated discrepancy (intentional or unintentional).

The patients themselves frequently generated medication discrepancies. Namely, 35.4% of all the included patients generated at least one unintentional discrepancy, with 19.3% of patients having at least one clinically important discrepancy. Some examples of clinically important discrepancies are presented in Supplementary Tables S4, S5. In the intervention group, significantly fewer patients experienced an unintentional patient-generated discrepancy (14.7% vs. 59.3%; *p* < 0.001) or a clinically important patient-generated unintentional discrepancy (8.1% vs. 32.2%; *p* < 0.001; Table 4). In the multiple logistic regression model, the intervention significantly reduced a patient’s risk of experiencing a clinically important patient-generated unintentional discrepancy by fivefold (OR 0.204, 95% CI 0.093–0.448; *p* < 0.001; Table 5), with no other factor showing a significant association.

**TABLE 5:** Multiple logistic regression results–clinically important patient-generated medication discrepancies 30 days after discharge.

Clinically important patient-generated medication discrepancies 30 days after discharge				
Covariates	Unintentional discrepancies		Intentional discrepancies	
	Nagelkerke’s R^2^ = 0.211; *p* = 0.761*		Nagelkerke’s R^2^ = 0.195; *p* = 0.683*	
	OR (95% CI)	*p-value*	OR (95% CI)	*p-value*
Gender (male vs. female)				
Male	Reference			
Female	0.751 (0.362–1.557)	0.441	0.297 (0.101–0.876)	**0.028**
Study group				
Control group	Reference			
Intervention group	0.204 (0.093–0.448)	**<0.001**	2.525 (0.843–7.563)	0.098
Age (years)	1.016 (0.984–1.049)	0.332	0.958 (0.923–0.993)	**0.020**
Charlson Comorbidity Index	1.000 (0.802–1.248)	0.999	1.026 (0.758–1.388)	0.868
Admission type				
Acute	Reference			
Planned	1.723 (0.721–4.117)	0.221	3.588 (0.987–13.037)	0.052
Reason for admission		0.164		**0.042**
Infection	Reference			
Malignancy	0.787 (0.181–3.431)	0.750	1.414 (0.163–12.252)	0.753
Heart disease	0.681 (0.210–2.211)	0.522	1.459 (0.313–6.814)	0.631
Respiratory disease	2.289 (0.916–5.722)	0.077	5.819 (1.597–21.205)	**0.008**
Other	1.246 (0.364–4.264)	0.727	1.394 (0.216–8.999)	0.727
Number of medicines at discharge	1.041 (0.935–1.159)	0.458	1.122 (0.972–1.294)	0.116

Abbreviations: OR, odds ratio; CI, confidence interval. Significant *p* values are marked in bold. *Hosmer-Lemeshow test.

More than 40% of patients deliberately deviated from discharge instructions for at least one medicine. In addition, almost 10% of all patients experienced at least one clinically important intentional patient-generated discrepancy (Table 4). Although not statistically significant, fewer patients in the intervention group experienced an intentional patient-generated discrepancy (36.8% vs. 47.5%; *p* = 0.085), with more experiencing a clinically important intentional patient-generated discrepancy (11.0% vs. 7.6%; *p* = 0.355). The intervention did not significantly increase the risk of a clinically important intentional patient-generated discrepancy (OR 2.525, 95% CI 0.843–7.563; *p* = 0.098). In the same model, the risk of at least one clinically important intentional patient-generated discrepancy was higher in men (OR 0.297, 95% CI 0.101–0.876; *p* = 0.028) and younger patients (OR 0.958, 95% CI 0.923–0.993; *p* = 0.020).The risk was also associated with the reason for admission (*p* = 0.042), particularly in the presence of respiratory disease (Table 5).

Altogether, a quarter of patients (26.3%) had at least one clinically important patient-generated discrepancy, either intentional or unintentional (Table 4).

## 4 Discussion

The present study reveals that patients undergo significant changes in their medicines within the first month after hospital discharge. Remarkably, a quarter of medicines were changed, with patients being responsible for half of these modifications, either intentionally or unintentionally. Furthermore, the study demonstrates that pharmacist-led medication reconciliation, coupled with patient counselling, reduces the risk of clinically important unintentional patient-generated discrepancies post discharge by fivefold. This outcome was achieved despite the intervention being delivered exclusively during the hospital stay, with no subsequent follow-up.

Hospitalization exposes patients to numerous changes in their medication regimens, not only during their stay but also shortly after discharge, as demonstrated in our study. Indeed, the majority of patients (81.5%) experienced at least one discrepancy within 30 days post discharge. Compared to the discharge letter, 25.3% of medicines were altered within the first month after discharge, which is consistent with findings from other studies (Mansur et al., 2008; Viktil et al., 2012). Moreover, these post-discharge modifications were more prevalent for medicines that had already been changed at the time of discharge. The rate of post-discharge changes was higher for medicines with unintentional discrepancies at discharge (59.3%) compared with those with intentional, documented discrepancies (23.5%).

Changes occurring after hospital discharge often reversed those implemented at the time of discharge. Specifically, over one-third of medicines (37.1%) that were omitted at discharge were reintroduced within the following 30 days. Our findings align with previous studies that emphasize the need for vigilance regarding alterations in medicine therapy in the first month following hospital discharge (Becker et al., 2021; Krause et al., 2020; Mansur et al., 2008; Viktil et al., 2012).

In our study, patients significantly influenced medication management after hospital discharge, accounting for half of the observed medication changes. The findings demonstrate that patients make changes to their medicines not only unintentionally but also intentionally. Specifically, over one-third of patients experienced an unintentional (35.4%) or an intentional (40.0%) patient-generated discrepancy. This finding contradicts the common simplification in the literature that changes made by physicians are considered intentional (Krause et al., 2020; Viktil et al., 2012), whereas all other changes are deemed unintentional (Marinović et al., 2021; Shiu et al., 2016). Further research should explore the reasons behind intentional patient-generated discrepancies, e.g., via qualitative interviews, to inform the design of future interventions and allied educational tools. A half of the unintentional and almost one-fifth of the intentional patient-generated changes were considered clinically important, with potential beneficial or detrimental effects. We intentionally refrained from categorizing potential consequences as harmful or beneficial, as they were often unclear in individual cases.

Numerous patient-generated discrepancies can be particularly concerning, especially when patients, often unintentionally, contradict an intentionally altered medicine in the discharge letter. Some of the most harmful and common examples of such changes include omission of digoxin that was newly introduced during hospitalization, continuation of treatment with a lower-than-recommended dose of beta-blockers in patients hospitalized for symptomatic atrial fibrillation, premature discontinuation of antibiotic therapy, continuation of treatment with metformin omitted due to metabolic acidosis, and multiplication of therapy with inhaled corticosteroids and bronchodilators. Conversely, in some instances, patients either intentionally or unintentionally did not follow a discharge recommendation involving an unintentional change in discharge therapy, thereby potentially preventing devastating consequences. An example of this is a patient who unintentionally omitted an erroneously increased dose of the immunosuppressant tacrolimus in the discharge letter and continued to take the same dose as before hospitalization.

Patient-generated changes were more common in certain groups of medicines: (i) those not related to the primary reason for hospitalization, such as medicines for acid-related disorders, and (ii) those that patients felt more confident adjusting for better symptom control, such as medicines for pulmonary diseases, analgesics, and sedatives. However, patients rarely altered groups of medicines considered high-risk for adverse events, such as antithrombotics.

A pharmacist-led medication reconciliation intervention at admission and discharge, coupled with patient counselling at discharge, significantly reduced the odds of a patient having a clinically important unintentional patient-generated discrepancy after hospital discharge by five-fold. This effect was not influenced by other patient or hospitalization characteristics evaluated in the multiple regression analysis, including sex, age, Charlson Comorbidity Index, reason for admission, admission type, and number of medicines at discharge. Furthermore, the results confirm the long-term benefits of preventing medication errors at hospital discharge. As we previously reported, the medication reconciliation intervention reduced the likelihood of a clinically important medication error by 20-fold, (Jošt et al., 2024). The current study demonstrates that such outcomes of medication reconciliation are crucial in reducing medication discrepancies after hospital discharge.

The study emphasizes the importance of the educational part of the intervention, particularly in the form of patient counselling at discharge. Due to sudden changes in patient’s health, physical, or cognitive state, medication management after hospitalization can present a significant challenge. Moreover, patients are likely to adhere to therapy better if they are more involved in decisions, which should also be well explained (Kardas, 2024). All patients in the intervention group benefited from counselling with additional written instructions in lay language on how to take medicines after discharge, empowering them to manage their medicines once back in their home environment.

It should also be noted that some patients may not able to manage their medicines independently after returning home. Therefore, information about medicine changes should be communicated not only to the patients but also to their caregivers in such cases (Mortelmans et al., 2021). Special attention should be paid to avoiding medication errors, particularly with high-risk medicines that may go unnoticed for extended periods of time after hospital discharge.

The intervention did not affect the rate of patients who intentionally changed their medicines, as some changes, especially those involving symptomatic medicines or medicines used for symptom relief, require proactive patient involvement.

This study highlights the importance of monitoring patients after hospital discharge due to the significant tendency of patients to alter their therapy, whether intentionally or unintentionally, which may result in harmful outcomes for patients (Weir et al., 2020). Patients often make these changes in response to symptoms or because of misunderstandings about their prescribed regimens. Our study shows the crucial role of pharmacists, closely integrated into the healthcare team and actively involved in transition of care in hospitals, on reduction of post-discharge therapy changes. In addition, pharmacists could support the education of patients about their therapy with enhanced individual counselling in close collaboration with GPs to ensure seamless medication management.

### 4.1 Strengths and limitations

Our study provides important insights into what happens after hospital discharge, as recent studies of medication reconciliation have provided valuable information on rehospitalizations or the occurrence of adverse events (Finlayson et al., 2018; Johansen et al., 2022; Kempen et al., 2021; Ravn-Nielsen et al., 2018), but not about the frequency and type of medication changes after hospital discharge.

Nevertheless, the study also has some limitations. In our study, data on patients’ medicines were collected 30 days after hospital discharge by telephone interviews with patients or their caregivers, whereas in most other studies of post-discharge medication discrepancies, information about patients’ medication regimen was obtained from interviews with GPs or data from local pharmacies (Krause et al., 2020; Marinović et al., 2021; Viktil et al., 2012). Information available to different healthcare professionals about a patient’s medication regimen after discharge may not be the most accurate if it is not cross-checked with the patient. We believe that focusing on the patient as the source of information, supported by data from the electronic prescribing system, increases the accuracy of medication regimen information. Also, directly interviewing patients enabled a better understanding of their role in medication management. This approach allowed patients to explain the background of their deviations from discharge therapy, which were categorized as intentional or unintentional. In particular, intentional patient-generated discrepancies are seldomly addressed in the literature. Additionally, we did not assess the actual consequences of the patient-generated discrepancies after discharge, which would provide better insight into the intentional and unintentional changes and enable further development/optimization of transition of care services. Moreover, in the study we did not focus on physician-generated discrepancies and the reasons for the medication changes they performed.

Our study showed that 40% of unintentional discrepancies made at discharge continued also after discharge. Due to methodology, similar to other studies, their clinical importance or consequences were not assessed as they were not considered post-discharge discrepancies. However, this limitation indicates the way for further research, in addition to the above-mentioned necessary focus on reducing unintentional discrepancies already present at discharge.

## 5 Conclusion

This study shows the importance of monitoring patients after their discharge from a hospital. Indeed, a quarter of the medicines were changed in the early post-discharge period, with half of these changes made either intentionally or unintentionally by the patients. In addition, pharmacist-led medication reconciliation coupled with patient counselling reduced the risk of clinically important unintentional patient-generated discrepancies after discharge by five-fold, while the rate of patients who intentionally changed their medicines was unaffected. The insight gained may help to develop future services that are better tailored to patients’ needs or further support patients in managing their medicines after hospitalization.

## Data Availability

The raw data supporting the conclusions of this article will be made available by the authors, without undue reservation.
