# Supplementary material for: Pharmacist-led hospital intervention reduces unintentional patient-generated medication discrepancies after hospital discharge
Source: Front Pharmacol. 2024 Oct 24;15:1483932. doi: 10.3389/fphar.2024.1483932 (PMC11551538; doi:10.3389/fphar.2024.1483932)
Supplement: Supplementary file 1 [file Table1.docx]

**Table S1:** Discrepancy type 30 days after discharge vs. at discharge (N=2441 medicines)

| *Discrepancy type at 30 days after discharge* | | | | | | |
| --- | --- | --- | --- | --- | --- | --- |
|  | | **No discrepancy** (N=1824) | **Change in dosage regimen** (N=187) | **Omission** (N=136) | **Addition** (N=294) | ***P value**** |
| *Discrepancy type at discharge* | **No discrepancy** (N=1113) | 1024 (92.0%) | 40 (3.6%) | 45 (4.0%) | NA | **<0.001** |
|  | **Change in dosage regimen** (N=235) | 142 (60.4%) | 82 (34.9%) | 11 (4.7%) | NA |  |
|  | **Omission** (N=396) | 249 (62.9%) | NA | NA | 147 (37.1%) |  |
|  | **Addition** (N=554) | 409 (73.8%) | 65 (11.7%) | 80 (14.4%) | NA |  |
|  | **Medicine not prescribed at discharge** (N=147) | NA | NA | NA | 147 (100.0%) |  |
| Abbreviations: NA – not applicable.  * Chi square test; significant p values are marked in bold. | | | | | |  |
