# Supplementary material for: Pharmacist-led hospital intervention reduces unintentional patient-generated medication discrepancies after hospital discharge
Source: Front Pharmacol. 2024 Oct 24;15:1483932. doi: 10.3389/fphar.2024.1483932 (PMC11551538; doi:10.3389/fphar.2024.1483932)
Supplement: Supplementary file 2 [file Table2.docx]

**Table S2**: Person generating discrepancy at 30 days after discharge vs. most common drug classes (N=2441 medicines)

| *Person generating discrepancy 30 days at discharge* | | | | | | | | | |
| --- | --- | --- | --- | --- | --- | --- | --- | --- | --- |
| Most common drug classes | | **No discrepancy** (N=1824) | | | **Physician** (N=294) | **Patient - intentional** (N=171) | **Patient - unintentional** (N=152) | ***P value**** | |
| Drugs for pulmonary diseases (R03) (N=365) | | 282 (77.3%) | 22 (6.0%) | | 31 (8.5%) | 30 (8.2%) | **<0.001** |  |  |
| Drugs for lowering HR and BP (C07, C08, C09) (N=362) | | 289 (79.8%) | 41 (11.3%) | | 14 (3.9%) | 18 (5.0%) |  |  |  |
| Antithrombotic drugs (B01) (N=179) | | 143 (79.9%) | 28 (15.6%) | | 2 (1.1%) | 6 (3.4%) |  |  |  |
| Analgesics (N02) (N=178) | | 113 (63.5%) | 34 (19.1%) | | 23 (12.9%) | 8 (5.5%) |  |  |  |
| Drugs for acid related disorders (A02) (N=154) | | 93 (60.4%) | 15 (9.7%) | | 17 (11.0%) | 29 (18.8%) |  |  |  |
| Sedatives, antidepressants and antipsychotics (N05, N06) (N=130) | | 98 (75.4%) | 12 (9.2%) | | 15 (11.5%) | 5 (3.8%) |  |  |  |
| Antidiabetics (A10) (N=108) | | 86 (79.6%) | 11 (10.2%) | | 8 (7.4%) | 3 (2.8%) |  |  |  |
| Diuretic in heart failure (C03C, C03D) (N=99) | | 72 (72.7%) | 18 (18.2%) | | 5 (5.0%) | 4 (4.0%) |  |  |  |
| Other (N=866) | | 648 (74.8%) | 113 (13.0%) | | 56 (6.5%) | 49 (5.7%) |  |  |  |
| * Chi square test; significant p values are marked in bold. | | | | | | | | | |
