# Supplementary material for: Pharmacist-led hospital intervention reduces unintentional patient-generated medication discrepancies after hospital discharge
Source: Front Pharmacol. 2024 Oct 24;15:1483932. doi: 10.3389/fphar.2024.1483932 (PMC11551538; doi:10.3389/fphar.2024.1483932)
Supplement: Supplementary file 3 [file Table5.docx]

**Table S 5**: Examples of clinically important intentional patient-generated discrepancies 30 days after discharge

| **Medicine** | **Description** |
| --- | --- |
| Short and long-acting insulin | In the discharge letter, insulin was instructed to be taken as prior to hospitalisation, without actual dosage regimen. After discharge, the patient increased insulin dose as he was experiencing hyperglycaemia, likely due to the concomitant introduction of dexamethasone. |
| Pantoprazole | In the discharge letter, pantoprazole (40 mg, od and prn, po) was omitted in a patient on continuous therapy with methylprednisolone. The change was unintentional. After discharge, the patient continued with pantoprazole regularly and prn in case of symptoms. |
| Metformin | In the discharge letter, metformin (850 mg, bid, po) was omitted in a patient with diabetes. The change was unintentional. After discharge, the patient continued with metformin because he did not get any instructions to stop it. |
| Empagliflozin | In the discharge letter, empagliflozin (10 mg, od, po) was omitted in a patient with heart failure. The change was unintentional. After discharge, the patient continued with empagliflozin because he did not get any instruction to stop it. |
| Bisoprolol | In the discharge letter, bisoprolol (2.5 mg od, po) was introduced in a patient admitted due to supraventricular tachycardia with numerous comorbidities. The change was intentional and documented. After discharge, the patient stopped the bisoprolol because he felt dizzy, tired, and sleepy. |
| Pravastatin | In the discharge letter, pravastatin (40 mg od, po) was introduced due to hypercholesterolemia. The change was intentional and documented. After discharge, the patient did not continue with pravastatin because he was unaware of its indication. |

Abbreviations: po, peroral; od, once daily; bid, twice daily; prn, per need.
