# Supplementary material for: Pharmacist-led hospital intervention reduces unintentional patient-generated medication discrepancies after hospital discharge
Source: Front Pharmacol. 2024 Oct 24;15:1483932. doi: 10.3389/fphar.2024.1483932 (PMC11551538; doi:10.3389/fphar.2024.1483932)
Supplement: Supplementary file 4 [file Table3.docx]

**Table S3**: Person generating discrepancy at 30 days after discharge vs. type of discrepancies at discharge (N=2441 medicines)

| *Person generating discrepancy 30 days after discharge* | | | | | | |
| --- | --- | --- | --- | --- | --- | --- |
|  | | **No discrepancy** (N=1824) | **Physician** (N=294) | **Patient-intentional** (N=171) | **Patient- unintentional** (N=152) | ***P value**** |
| *Type of discrepancies at discharge* | **No discrepancy** (N=1113) | 1024 (92.0%) | 50 (4.5%) | 26 (2.3%) | 9 (0.8%) | **<0.001** |
|  | **Intentional, documented** (N=659) | 504 (76.5%) | 62 (9.4%) | 49 (7.4%) | 44 (6.7%) |  |
|  | **Intentional, undocumented**** (N=280) | 196 (70.0%) | 24 (8.6%) | 27 (9.6%) | 33 (11.8%) |  |
|  | **Unintentional, undocumented **** (N=246) | 100 (40.7%) | 24 (9.8%) | 61 (24.8%) | 61 (24.8%) |  |
|  | **Medicine not prescribed at discharge** (N=147) | NA | 134 (91.2%) | 8 (5.6%) | 5 (8.3%) |  |
| Abbreviations: NA – not applicable.  * Chi square test; significant p values are marked in bold.  ** Intentional documented and unintentional discrepancies together defined as medication error at discharge. | | | | | | |
