# Supplementary material for: Pharmacist-led hospital intervention reduces unintentional patient-generated medication discrepancies after hospital discharge
Source: Front Pharmacol. 2024 Oct 24;15:1483932. doi: 10.3389/fphar.2024.1483932 (PMC11551538; doi:10.3389/fphar.2024.1483932)
Supplement: Supplementary file 5 [file Table4.docx]

**Table S 4**: Examples of clinically important unintentional patient-generated discrepancies 30 days after discharge

| **Medicine** | **Description** |
| --- | --- |
| Tacrolimus | In the discharge letter, tacrolimus dose was increased (from 2.5 mg od, po, to 3.5 mg od, po). The change was unintentional. After discharge, the patient was unaware of the change and continued tacrolimus as prior to hospitalisation (2.5 mg od, po). |
| Fluticasone / vilanterol AND formoterol / beclomethasone | In the discharge letter, fluticasone/vilanterol was changed to formoterol/beclomethasone due to patient’s difficulties with handling first device. The change was intentional but undocumented. After discharge, the patient did not know about the reason for the changes and was taking both medicines concomitantly. |
| Naproxen AND  diclofenac | In the discharge letter, diclofenac (75 mg prn, po) was added to drug therapy and no instruction were given about naproxen, used by the patient (550 mg prn, po) before hospitalisation. The change was unintentional. After discharge, patient continued with both medicines in high doses, prn, few times weekly. |
| Perindopril / indapamide / amlodipine | In the discharge letter, perindopril (4 mg od, po, uptitrated according to blood pressure) was prescribed instead of perindopril / indapamide / amlodipine (8 / 2.5 / 10 mg od, po) due to low BP and hypokalaemia. The change was intentional and documented. After discharge, the patient was not aware of the change and continued with perindopril/indapamide/amlodipine. |
| Ramipril | In the discharge letter, ramipril (5 mg od, po) was discontinued due to hyperkalaemia. The change was intentional and documented. After discharge, the patient recalled one antihypertensive medicine being omitted but was unaware of which one specifically, thus continued ramipril and stopped taking lacidipine. |
| Digoxin | In the discharge letter, digoxin (0.1 mg od, 5 times weekly, po) was introduced due to tachycardia. The change was intentional and documented. After discharge, the patient was unaware of the need to continue with digoxin post discharge and was not taking it. |
| Furosemide | In the discharge letter, furosemide (20 mg od, po) was introduced due to symptomatic heart failure and hyperkalemia. The change was intentional and undocumented. After discharge, the patient was unaware on the need to take furosemide regularly and stopped it few days after discharge. |
| Trimetazidine | In the discharge letter, trimetazidine (35 mg bid, po) was introduced due to angina pectoris. The change was intentional and documented. After discharge, the patient was unaware of the change and stopped with trimetazidine. |
| Amoxicillin / clavulanic acid | In the discharge letter, amoxicillin / clavulanic acid (875 mg/125 mg bid, po, for 7 days) was prescribed due to acute bronchitis. The change was intentional and documented. After discharge, the patient was unaware of the change and was not taking the antibiotic. |

Abbreviations: po, peroral; od, once daily; bid, twice daily; prn, per need.
